# Use of Artificial Intelligence in the Diagnosis of Colorectal Cancer

**DOI:** 10.7759/cureus.53024

**Published:** 2024-01-26

**Authors:** Basil N Nduma, Stephen Nkeonye, Tesingin D Uwawah, Davinder Kaur, Chukwuyem Ekhator, Solomon Ambe

**Affiliations:** 1 Internal Medicine, Medical City, Denton, USA; 2 Oncology, University of Texas MD Anderson Cancer Center, Houston, USA; 3 Internal Medicnie, Cherubin Family Health Care, Brooklyn, USA; 4 Internal Medicine, Medical City, North Richland Hills, USA; 5 Neuro-Oncology, New York Institute of Technology College of Osteopathic Medicine, Old Westbury, USA; 6 Neurology, Baylor Scott & White Health, McKinney, USA

**Keywords:** colorectal cancer (crc), machine learning, screening, disease diagnosis, effectiveness, efficacy, accuracy, sensitivity, specificity, artificial intelligence (ai)

## Abstract

Colorectal cancer (CRC) is one of the most common forms of cancer. Therefore, diagnosing the condition early and accurately is critical for improved patient outcomes and effective treatment. Recently, artificial intelligence (AI) algorithms such as support vector machine (SVM) and convolutional neural network (CNN) have demonstrated promise in medical image analysis. This paper, conducted from a systematic review perspective, aimed to determine the effectiveness of AI integration in CRC diagnosis, emphasizing accuracy, sensitivity, and specificity. From a methodological perspective, articles that were included were those that had been conducted in the past decade. Also, the articles needed to have been documented in English, with databases such as Embase, PubMed, and Google Scholar used to obtain relevant research studies.

Similarly, keywords were used to arrive at relevant articles. These keywords included AI, CRC, specificity, sensitivity, accuracy, efficacy, effectiveness, disease diagnosis, screening, machine learning, area under the curve (AUC), and deep learning. From the results, most scholarly studies contend that AI is superior in medical image analysis, the development of subtle patterns, and decision support. However, while deploying these algorithms, a key theme is that the collaboration between medical experts and AI systems needs to be seamless. In addition, the AI algorithms ought to be refined continuously in the current world of big data and ensure that they undergo rigorous validation to provide more informed decision-making for or against adopting those AI tools in clinical settings. In conclusion, therefore, balancing between human expertise and technological innovation is likely to pave the way for the realization of AI's full potential concerning its promising role in improving CRC diagnosis, upon which there might be significant patient outcome improvements, disease detection, and the achievement of a more effective healthcare system.

## Introduction and background

Considering public health, one common disease that has continually taken a toll on patients' health-related quality of life is colorectal cancer (CRC). By 2020, new cases of CRC were about 1.93 million - as reported by the International Agency for Research on Cancer, a statistic that placed the disease as the third most common cancer [1]. It is also notable that the incidence and prevalence of CRC are more pronounced in countries undergoing social and economic transition. Furthermore, apart from the risk factors of unhealthy lifestyle, obesity, and smoking, CRC incidence has been associated with family factors, genetic causes, and gender [2]. Indeed, the leading diagnostic approaches currently employed for CRC include histopathology examination, imaging, endoscopy, and laboratory tests [3]. Yet, the incidence of the disease continues to increase even in the wake of such tools.

Artificial intelligence (AI) is a concept that revolves around the study of human intelligence activity principles, upon which artificial systems could be developed, a process followed by examining how computers could be utilized in completing tasks that would previously call for input from human intelligence [4]. AI technologies, with a specific emphasis on machine learning (ML) and deep learning (DL), have advanced rapidly in the field of medical care, offering new potential for developing accurate and powerful computer-assisted techniques through which cancer could be screened, diagnosed, and treated effectively, upon which the patient prognosis could be followed [5].

Recently, more and more scholarly investigations have applied AI in the CRC field [6-8]. Important to remember is that conventionally, from the perspective of CRC screening, endoscopy has been considered the gold standard for screening individuals for CRC. The procedure tends to be supplemented with a fecal occult blood test (FOBT). Yet, these approaches are relatively determined by (or dependent on) experience, a flaw that suggests further that they remain prone to misdiagnoses and omissions. Also, in the current world of big data, an increase in electronic medical records (EMRs) and endoscopic imaging datasets implies that new and seamless approaches might be feasible to ensure convenience and error reduction. Here, the utilization of high-risk prediction models through omics and clinical data and the exploitation of AI-assisted endoscopy to detect and characterize polyps might steer improvements in CRC screening efficiency and accuracy [7].

From the diagnostics perspective, CRC's qualitative diagnosis and staging rely on pathological examination and radiography. With the field of image recognition experiencing advancements in processing technology, the need to reduce misdiagnosis rates, eliminate variations in experience, and improve medical image readability could not be overstated [8]. This review paper aims to comprehensively analyze and summarize the clinical value of AI technologies and research progress in CRC screening and diagnosis. The motivation of the study is to yield a complete picture of the current pattern of AI usage in essential clinical procedures of CRC diagnosis, particularly concerning the effectiveness or efficacy of the technologies and their associated accuracy.

A few factors explain the motivation behind this study. One of them is that if left untreated, CRC could lead to the adversity of increased annual healthcare costs at the individual, family, and national levels, hence the need to reverse this situation by discerning the efficacy of AI in CRC diagnosis (or otherwise) and, thus, proceeding to contribute to the current state-of-the-art via the provision of recommendations as deemed appropriate. The second motivation is that AI utilization, if ascertained to be effective, might go a long way in contributing positively to the reduction of the length of stay at the hospital on the part of patients, with an associated tertiary effect being a marked improvement in health-related quality of life. Thirdly, the study has been motivated by the criticality of ensuring that problems such as misdiagnoses arising from reliance on the providers' expertise are mitigated, hence yielding secondary benefits such as reductions in mortality rates and patient dissatisfaction with services. What is also worth remembering is that the study strives to give insight into any challenges or limitations that could be associated with the clinical implementation of AI, a pattern projected to pave the way for the description of any needed efforts for resolving such issues to ensure seamless adoption and implementation of the AI application in medicine.

## Review

Materials and methods

This was a review paper, so specific databases were used to collect secondary data. Some of them included Embase, PubMed, and Google Scholar. The search query saw some keywords relied on. Some had AI, CRC, specificity, sensitivity, accuracy, efficacy, effectiveness, disease diagnosis, screening, deep learning, area under the curve (AUC), and machine learning. The inclusion criterion was that the articles used in the investigation must have been developed in English. In addition, the dates of publication of studies that may have been cited in the selected articles were not restricted. However, the articles to be included in the investigation needed to have been developed in the last decade to ensure reliance on more recent research articles that would be deemed relevant to the current state-of-the-art concerning CRC diagnosis in the wake of a growing role of technology. The need to focus on eligible articles only prompted a review of references in the selected articles, implying that there was an independent review of the respective research studies by the research team participants to confirm that the studies satisfied the inclusion criterion. Some articles deemed less helpful and considered for exclusion from this review included conference abstracts, preclinical studies, commentaries, and those with inaccessible full texts. Also, articles written in a non-English language were excluded from the review paper.

**Figure 1:**
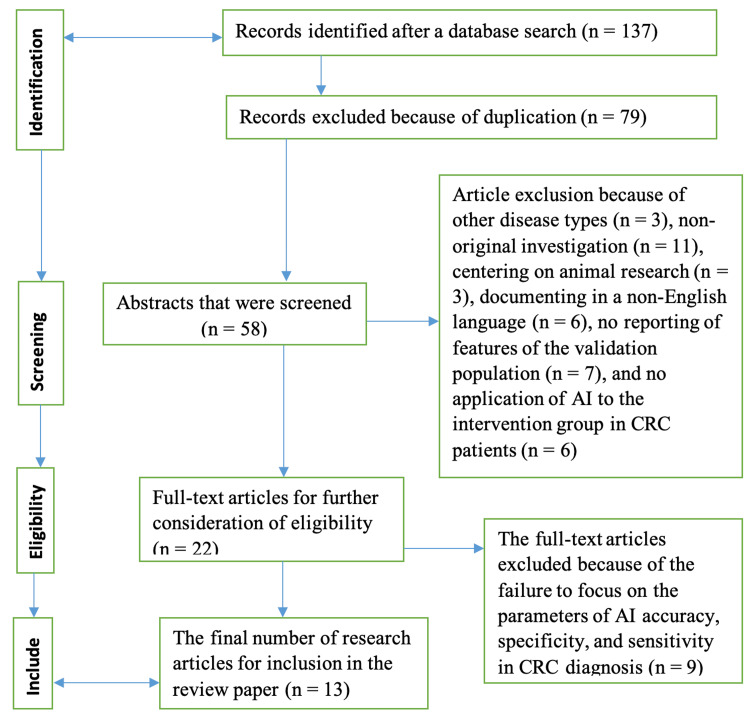
A summary of the article selection process

Regarding the research studies that satisfied the inclusion criterion and were included in the review, the critical information extracted had the population studied, their demographic features, controls and intervention group details, and the research designs. The figure below demonstrates how articles were screened, included, or excluded from the study. Figure 1 below shows a summary of the article selection process.

Results

Table 1 below outlines the results containing included studies with citations. With groundbreaking research, great application potential comes with the utilization of AI in various CRC clinical aspects. The study concluded, thus, that AI enables clinicians to conduct certain crucial tasks, including predicting survival and recurrence, therapeutic assessment, CRC qualitative and staging diagnosis, and the detection of colorectal polyps [9].

**Table 1:** Results containing included studies and citations CRC: Colorectal cancer; AI: Artificial intelligence; ML: Machine learning; DL: deep learning; AUC: area under the curve: cfDNA: Circulating free DNA; ANN: Artificial neural network; CP-ANN: counter propagational ANN; EC-CAD: endocytoscopy computer-aided diagnosis; PIVI: Preservation and Incorporation of Valuable Endoscopic Innovations; SVM: support vector machine

Reference	Author(s)	Year of Publication	Aim of Study	Number of Participants/Dataset Items	Results	Clinical Implications
[9]	Qiu et al.	2022	To determine the effectiveness of AI in CRC prognosis, treatment, screening, and diagnosis.	109	With groundbreaking research in ML, particularly DL, a great application potential comes with the utilization of AI in various CRC clinical aspects. The study concluded, thus, that AI enables clinicians to conduct certain crucial tasks, including predicting survival and recurrence, therapeutic assessment, CRC qualitative and staging diagnosis, and the detection of colorectal polyps.	In clinical environments, the potential of AI in enhancing the accuracy of CRC diagnosis is vivid, but identifying candidates for this application demands comprehensive expertise engagement.
[10]	Wan et al.	2019	To conduct a retrospective study and investigate the role of AI in detecting cfDNAs in CRC patients	271 non-cancer controls and 546 CRC individuals	In the findings, the area under the curve (AUC) was 0.92, with the ANN, which was used as the AI type, yielding a specificity value of 85% and sensitivity of 85%.	The results demonstrated the promising role of AI in CRC diagnosis due to high specificity and sensitivity values.
[11]	Peng et al.	2016	To conduct a prospective study in assessing AI’s potential in diagnosing and staging CRC	117	In the results, ANN yielded a relatively high accuracy value of 87.9% and a very high specificity value of 97.8%. However, the sensitivity value was significantly lower than the aforementioned parameters, standing at 53.8%.	High accuracy and high specificity suggested that AI is a reliable direction for diagnosing and staging CRC, but the need to ascertain its sensitivity by focusing on different clinical conditions, environmental conditions, and varying patient demographic features remains vivid.
[12]	Zhang et al.	2019	To implement a prospective study aimed at understanding CRC diagnosis and its associated genetic mutations.	312 tissue samples	With the counter propagational ANN (CP-ANN) used as an AI type, the accuracy, sensitivity, and specificity values that were established stood at 93.8%, 100%, and 87.5%, implying that the efficacy of AI usage in CRC staging, diagnosis, and prediction of potential recurrence was ascertained.	The superiority of AI in CRC diagnosis proved vivid, with very high accuracy depicting its potential in countering misdiagnoses and other errors that would otherwise emerge in clinical contexts following overdependence on the clinical expertise of providers.
[13]	Akbari et al.	2018	To conduct a retrospective study examining the effectiveness and accuracy of AI in segmenting CRC polyps.	200 images	Using a convolutional neural network (ANN), the study revealed very high accuracy and sensitivity and accuracy values of 99.3% and 97.7%, respectively. However, specificity was relatively low, standing at 74.8%.	With high accuracy and sensitivity, the study depicted the need to embrace AI in clinical environments relative to the segmentation of polyps. However, factors explaining lower specificity and how to mitigate them via possible redesigning of the CNN network as the AI type is an area worth exploring even further.
[14]	Takeda et al.	2017	To conduct a retrospective study and investigate the accuracy of computer-aided diagnosis (CAD) for invasive CRC using endocytoscopy images.	375 lesions	Through endocytoscopy CAD (EC-CAD) as a selected AI tool, this study revealed a specificity value of 98.9%, as well as 89.4% as the sensitivity value and 94.1% as the accuracy value, leading to a conclusion that AI exhibits superiority relative to CRC diagnosis.	The study suggested that with EC-CAD exhibiting promising values of sensitivity, accuracy, and specificity, identifying candidates for this procedure is key relative to the needed parameters before implementing the diagnostic procedure, hence optimal patient outcomes.
[15]	Blanes-Vidal et al.	2019	To determine the efficacy of integrating AI algorithms in CRC screening and diagnosis.	225	Compared to previous techniques, the autonomous detection algorithm yielded unprecedented high specificity, sensitivity, and accuracy, standing at 93.3%, 97.1%, and 96.4%, respectively.	The results suggested that through a matching algorithm, the similarity between colonoscopy polyps and colorectal capsule endoscopy could be quantified objectively, hence the promising role depicted by the autonomous detection algorithm, a pattern also worth embracing in clinical practice contexts.
[16]	Kominami et al.	2016	This study sought to uncover the effectiveness of a newly developed real-time image recognition system in supporting colorectal lesion diagnosis via narrow-band imaging.	41	Following the implementation of a real-time image recognition system through a support vector machine (SVM) algorithm, the researchers reported values of specificity, sensitivity, and accuracy as 93.3%, 93.0%, and 93.2%.	The findings demonstrated that through a real-time image recognition system, the Preservation and Incorporation of Valuable Endoscopic Innovations (PIVI) recommendations could be satisfied about colorectal tumor diagnosis, but the need for further investigation to ascertain these findings in related clinical contexts or with different patient groups with also varying demographic features is key.
[17]	Kudo et al.	2020	Through a multi-center study, the authors strived to establish EndoBRAIN’s diagnostic accuracy in colon neoplasm identification.	69,142 images	The value of sensitivity with which the EndoBRAIN identified colon lesions stood at 96.9%, with the algorithm’s associated specificity and accuracy values being 100% and 98%, respectively.	Through EndoBRAIN, neoplastic lesions could be differentiated from non-neoplastic lesions in endocytoscopic narrow-band images and stained endocytoscopic images.
[18]	Wang et al.	2018	The investigation aimed to develop a deep-learning algorithm and assess its effectiveness in polyp detection relative to clinical colonoscopies	1,290	Results demonstrated a sensitivity value of 94.38%, with the specificity value standing at 95.92% and the area under the curve 0.984.	The study indicated that AI may assist endoscopists during colonoscopy through a relatively accurate assessment of differences in adenoma and polyp detection performance.
[19]	Mori et al.	2015	The authors’ aim involved developing and evaluating the effectiveness of a novel computer-aided diagnosis system in colorectal lesion endocytoscopic imaging.	152	The novel computer-aided diagnosis system yielded accuracy and sensitivity values of 89.2% and 92.0%, respectively. However, the specificity value was relatively lower than the first two parameters documented above, standing at 79.5%.	The novel computer-aided diagnosis system proved to be relatively superior in performance but more research efforts ought to be embraced to understand factors leading to its relatively lower specificity value, hence the eventual informed decision-making process for or against embracing this AI tool.
[20]	Misawa et al.	2018	To determine the level of performance of a proposed model of AI-assisted CRC diagnosis retrospectively.	546	Findings demonstrated the accuracy, specificity, and sensitivity values of 75.5%, 63.3%, and 90.0%.	Whereas high sensitivity was obtained, the proposed algorithm yielded relatively lower accuracy and specificity values. In the future, it becomes relevant to discern the key factors accounting for these variations, with the specificity and accuracy values also found to be significantly lower than the values reported in other studies documented in these results.
[21]	Song et al.	2020	To develop and implement a computer-aided diagnostic system for CRC diagnosis through deep-learning technology, as well as validate the performance of the system.	12,480 images	With assistance from computer-aided diagnosis, there was an increase in the overall diagnostic accuracy from a range of 63.8% to 71.8% to a range of 82.7% to 84.2%.	Through the use of a deep-learning model, computer-aided diagnosis could lead to an accurate assessment of polyps, hence facilitating endoscopists’ diagnosis of colorectal polyps, if any.

Discussion

In one of the recent scholarly investigations, 103 articles were analyzed in a systematic review, with the aim of discerning the effectiveness of AI and its potential for screening, diagnosing, and staging CRC [9]. The results of this study demonstrated that through AI, clinicians could conduct certain tasks with accuracy and precision, including the prediction of recurrence and survival, therapeutic assessment, detection of colorectal polyps, and staging of CRC. It should be noted that although AI plays a promising role in steering the accurate diagnosis of CRC, some patients may be exhibiting pre-existing conditions, including those impacting the gastrointestinal system, and it is unclear whether or not such factors could impair the accuracy of AI in CRC diagnosis. In another study, the artificial neural network (ANN) was evaluated to determine its effectiveness in CRC diagnosis, with 271 non-cancer individuals in the control group and 546 CRC cases in the test group [10]. Conducted from a retrospective perspective, this study sought to inform the clinical application of AI, the implications for the current state-of-the-art, and any potential for errors. In the study result, high sensitivity and specificity values were produced by ANN as the AI type, standing at 85% each. As such, the study confirmed the efficacy of AI usage in CRC diagnosis. In this study, there was a marked variation in the demographic features of the study participants. As such, the study's larger sample size might have contributed to outcome reliability. The aforementioned variation in the participant demography and any impact it might have posed on the accuracy of the ANN application remained unexplained. Still, with ANN as the focus, another review in this article is a prospective study conducted to ascertain the role of ANN in CRC staging and diagnosis [11]. With 117 participants in the study, the aim was to obtain the accuracy, sensitivity, and specificity values of this type of AI, thus making informed inferences concerning its potential for CRC diagnosis. In the results, the study depicted the accuracy, sensitivity, and specificity values as 87.9%, 53.8%, and 97.8%, respectively. As such, it was inferred that ANN comes with relatively high accuracy and very high specificity. The sensitivity value was much lower than the values of the other two parameters. The factors that might have led to this potential flaw in ANN remained unknown. Whether the study's design, the number of participants, or the clinical and environmental conditions might have impaired the accuracy of ANN was not documented vividly. Despite this potential flaw, the authors concluded that AI continues to pose a promising role in enhancing CRC diagnosis and staging, with the tertiary benefit projected to entail significant reductions in errors, having mitigated overreliance on human expertise [11].

In a prospective study, the efficacy of AI in CRC staging, diagnosis, and prediction of probable recurrence was investigated [12]. In this study, the central motivation was to shed light on the extent of accuracy of the counter-propagational ANN (CP-ANN) following its implementation in achieving the aforementioned study aim. In the results, this type of AI achieved a very high accuracy value of 100%, with specificity and sensitivity values also standing high at 87.5% and 100%, respectively. Indeed, the key strength of this study was that a relatively large sample size of the selected tissue samples was utilized, hence boosting the aspects of outcome validity and reliability. However, it should be noted that in such a large sample, the disease stage of CRC might differ from individual to individual. Therefore, whether the stage and severity of the disease might have impacted the specificity and sensitivity performance of CP-ANN remained unclarified.

In a retrospective study, the CNN network was used as an AI type to investigate its effectiveness in segmenting polyps alongside CRC diagnosis [13]. With 200 images analyzed, the study strived to highlight the extent to which AI could contribute to and improve human expertise in clinical contexts, hence its potential use for future disease diagnosis. The findings reported very high sensitivity and accuracy values, which stood at 99.3% and 97.7%, respectively. However, a relatively low specificity value was obtained, reported to be 74.8%. As such, it can be inferred that the researchers obtained mixed outcomes, but high accuracy implied that AI could assist in diagnosing CRC reliably. It is worth noting that some conditions may impact the accuracy of CRC screening, including care fragmentation and a lack of follow-up. In this case, therefore, how the CNN network interacts with these parameters and whether or not the parameters might impair its accuracy are research directions that are yet to receive clarity.

Computer-aided diagnosis (CAD) is another type of AI tool used to determine the effectiveness of AI technology in CRC diagnosis in one of the reviewed studies is CAD [14]. With endocytoscopy CAD (EC-CAD) on focus, 375 lesions were examined, and the findings portrayed highly promising outcomes regarding using AI in disease diagnosis. The specificity, sensitivity, and accuracy values were 98.9%, 89.4%, and 94.1%, respectively. These results led to an inference that AI plays an increasing role in disease diagnosis for CRC. On the one hand, the key strength of the study involved reliance on a larger number of sampled lesions. On the other hand, the study was retrospective, pointing to the possibility of difficulty in assessing the temporal relationship. Whether the AI tool might have been prone to this weakness remained unknown.

To give further insight into this subject, a deep CNN algorithm for the localization and autonomous detection of colorectal polyps was examined alongside a new algorithm for matching colonoscopy polyps and colorectal capsule endoscopy (CCE) relative to objective measures of similarity between polyps [15]. In this study, which was fully paired and conducted between 2015 and 2016, involving 255 participants drawn from a Danish national screening program, the motivation was to present two innovative data science algorithms through which there could be a considerable improvement in relevant data acquisition and analysis. In the findings, the authors noted that away from previous methods of matching CCE and colonoscopy polyps, the proposed matching algorithm could objectively quantify the similarity between colonoscopy polyps and CCE based on their location, morphology, and size. The algorithm was also affirmed to offer a one-to-one equivocal match between colonoscopy polyps and CCE. Indeed, unprecedented high specificity, sensitivity, and accuracy values were obtained through the implementation of the autonomous detection algorithm, with the values of these parameters standing at 93.3%, 97.1%, and 96.4%, respectively. With a relatively large sample size focused, it can be inferred that the researchers reported promising outcomes that could be deemed valid and reliable. However, it is worth acknowledging that the distribution and severity of colorectal polyps will be expected to vary from individual to individual. At this point, whether these parameters might have impacted the performance of the autonomous detection algorithm remained undocumented.

The extent to which a newly developed real-time image recognition system could aid in Colorectal lesion diagnosis forms an additional area that has received scholarly attention [16]. The latter study aimed to give insight into how real-time image recognition system analysis could compare with the outcomes yielded by narrow-band imaging diagnosis, eventually allowing informed decision-making concerning the correlation between pathological results and image analysis. The selected study focused on 41 patients who had undergone endoscopic resection of 118 colorectal lesions, 73 of them being neoplastic lesions and 45 non-neoplastic lesions. Importantly, the researchers contended that relative to the need to satisfy some problems with the recommendations by the Preservation and Incorporation of Valuable Endoscopic Innovations (PIVI) committee, the need for a new direction in AI-assisted CRC screening could not be overemphasized. In the results, concordance between diagnosis by real-time image recognition system with an SVM algorithm and endoscopic diagnosis yielded an output value of 97.5%, with the specificity, sensitivity, and accuracy values obtained as 93.3%, 93.0%, and 93.2%, respectively. At this point, the aforementioned high values for the three parameters suggested that the proposed real-time image recognition system remained promising in satisfying the PIVI recommendations, proving also reliable in supporting colorectal tumor diagnosis. However, it is worth noting that with 41 patients on the focus, there remains a need for further research to center on larger populations with varying demographic features and health condition characteristics before conclusively affirming the performance of the real-time image recognition system. To identify colorectal neoplasms, the effectiveness of the EndoBRAIN was investigated, aimed at shedding light on the extent to which AI could contribute to CRC diagnostic outcome accuracy or superiority [17]. In this study, the authors noted that through precise colorectal polyp diagnosis, colonoscopy's cost-effectiveness could be improved markedly, as well as reduce complications that may be associated with polypectomy. The authors acknowledged further that obtaining adequate diagnostic performance remains a challenge for community-based non-experts. As such, AI-based systems have emerged in a quest to ensure superior endoscopic image analysis and identify neoplasms with low inter-observer variation and high accuracy. Motivated by the need to contribute to this current state-of-the-art, the researchers aimed to perform a multi-center investigation to uncover EndoBRAIN's diagnostic accuracy. Notably, EndoBRAIN reflects an AI algorithm for analyzing micro-vessels, crypt structure, and cell nuclei in endoscopic images, upon which the presence or absence of colon neoplasms could be discerned.

From a methodological perspective, the authors trained the EndoBRAIN system through 69,142 images. In turn, a retrospective comparative analysis of EndoBRAIN's diagnostic performance was performed, and the results were compared with those reported by 30 endoscopists, who constituted 10 experts and 20 trainees. As mentioned earlier, the chief intention was to establish the level of performance of EndoBRAIN in assessing endocytoscopic images, especially in terms of the algorithm's accuracy in the distinction of non-neoplasms from neoplasms. In the results, the ability of EndoBRAIN to identify colon lesions relative to stained endocytoscopic images came with 98% accuracy, 100% specificity, and 96.9% sensitivity. Indeed, the values were avowed to be significantly greater than those reported by endoscopy experts and trainees. When it came to narrow-band image analyses, EndoBRAIN distinguished non-neoplastic images from neoplastic images with 96.0% accuracy, 94.3% specificity, and 96.9% sensitivity [17]. Notably, these values remained significantly superior to or higher than those endoscopy trainees had reported, but they would remain comparable to those endoscopy experts reported. As such, it was concluded that the EndoBRAIN technology could be implemented in clinical contexts where small polyps could be evaluated endoscopically. However, even as the study exhibited superiority whereby the sample size of the chosen images was very large, its key weakness was that the number of endoscopy trainees whose results were compared when implementing the AI algorithm was double the number of experts. At this point, whether similar performance outcomes of the AI tool could be obtained if these numbers are altered or the findings might not be significantly difference is an area in need of more and more scholarly investigation and clarification.

To shed additional light on the interplay between AI and CRC diagnosis, a validation deep learning algorithm was developed to discern its efficacy in polyp detection during colonoscopy [18]. The motivation of the latter study accrued from a position that whereas a gold standard for colon cancer prevention is through precancerous polyp detection and removal, the rate at which adenomatous polyps are detected tends to vary considerably from one endoscopist to another. As such, the investigation strived to examine the effectiveness of a machine-learning algorithm in polyp detection relative to clinical colonoscopies. Particularly, how real-time the algorithm could be was the intended objective, especially by obtaining the area under the curve and specificity and sensitivity values. The findings established that based on the data obtained from 1,290 patients, the area under the curve was 0.984, while the specificity and sensitivity values were 95.92% and 94.38%, respectively. These promising outcomes, thus, demonstrated that through the implementation of an otherwise multi-threaded processing system, the algorithm on the focus could process 25 or more frames per second with a promising latency value when implementing real-time video analyses of CRC diagnoses. Hence, the resulting AI software could pave the way for superior colonoscopy performance among endoscopists and aid in assessing differences in adenoma and polyp detection performance.

In the context of a university hospital, a novel computer-aided diagnosis system was developed and evaluated for colorectal lesion endocytoscopic imaging [19]. Here, the authors obtained data from 152 patients to discern the accuracy, specificity, and sensitivity values of the diagnosis system relative to its role in CRC diagnosis, if any. Indeed, the results demonstrated high accuracy and sensitivity values of 89.2% and 92.0%, respectively. However, there was a relatively lower specificity value, which was 79.5%. As such, it can be inferred that mixed outcomes were obtained, and in the future, it becomes critical to investigate the proposed algorithm even more comprehensively to discern the key factors that might explain the lower specificity value(s). In so doing, a more informed decision for or against adopting the AI tool might arise.

AI-assisted polyp detection has also been implemented using short colonoscopy videos to determine the extent of performance superiority, if any [20]. In the selected study, 546 short colonoscopy videos were focused on, with polyp-negative videos being 391 and polyp-positive videos being 155. The proposed model's accuracy, specificity, and sensitivity values in the retrospective study stood at 76.5%, 63.3%, and 90.0%. As such, a high sensitivity value was obtained, but accuracy and specificity values were relatively lower. The values were also significantly lower than those reported in the earlier studies. Indeed, the key strength of the study is that it relied on a larger number of colonoscopy videos, hence sample size adequacy. However, its major weakness was that the key parameters explaining lower specificity and accuracy values remained unknown. Also, the inferior performance of the proposed algorithm to the majority of algorithms employed in other research studies documented above was not clarified, yielding a dilemma on whether to recommend using the AI tool in clinical contexts during CRC diagnostic testing and screening.

Recently, a computer-aided diagnostic system (CAD) was also developed, and its performance was validated using deep-learning technology [21]. With 12,480 images analyzed, values ranging from 81.3% to 82.4% were obtained as the overall diagnostic accuracy of CAD. These values were documented to be comparable to those of experts and higher than the trainees. Also, it was noted that there was an increase in the overall diagnostic accuracy from a range of 63.8% to 71.8% to a range of 82.7% to 84.2%. From these findings, it was inferred that CAD, upon employing a deep-learning model, could yield accurate polyp assessment, suggesting further that the AI tool could facilitate colorectal polyp diagnosis. However, even when the key strength of the study entailed reliance on a very large sample of image patches, it is evident that the accuracy was relatively moderate compared to the accuracy values obtained by most authors documented above. Therefore, In the study, how to improve the CAD system to ensure very high accuracy values remained unknown, requiring further insight.

## Conclusions

In summary, CRC reflects one of the most debilitating health conditions a larger population grapples with. Recently, scholarly attention has been drawn to the diagnosis of this condition, a direction representing a significant advancement in medical technology. In this review paper, most of the primary studies consulted contend that AI has demonstrated its potential to enhance efficiency and accuracy in CRC detection and diagnosis via data analysis and imaging techniques. With machine learning algorithms and deep learning models, leveraged AI can aid in analyzing medical images, develop subtle patterns, and enable healthcare professionals to make more informed decisions. However, even at a time when AI holds great promise, it is worth acknowledging that as the algorithms are deployed, there is a seamless collaboration between medical experts and AI systems, as well as continuous refinement of the algorithms and their rigorous validation. In summary, striking a balance between human expertise and technological innovation might go a long way in ensuring that the full potential of AI is realized in its improvement of the diagnosis of CRC, with future clinical environments poised to be marked by improved patient outcomes, earlier detection, and a more effective healthcare system.
